# Author Correction: Multifunctional nanoagents for ultrasensitive imaging and photoactive killing of Gram-negative and Gram-positive bacteria

**DOI:** 10.1038/s41467-025-61902-y

**Published:** 2025-07-17

**Authors:** Jiali Tang, Binbin Chu, Jinhua Wang, Bin Song, Yuanyuan Su, Houyu Wang, Yao He

**Affiliations:** Laboratory of Nanoscale Biochemical Analysis, Institute of Functional Nano and Soft Materials (FUNSOM), Soochow University, Suzhou, 215123 China

Correction to: *Nature Communications* 10.1038/s41467-019-12088-7, published online 6 September 2019

In the version of the article initially published, in the “HeLa” panel in Fig. 7b, the “GP-Ce6-SiNPs, control group” image was a duplicate of the “GP-Ce6-SiNPs, group 2” image and the “Ce6, group 3” image was a duplicate of the “SiNPs, group 3” image. The corrected Fig. 7b, HeLa panel can be found below as Fig. 1.

Fig. 1 Corrected Fig. 7b, HeLa.
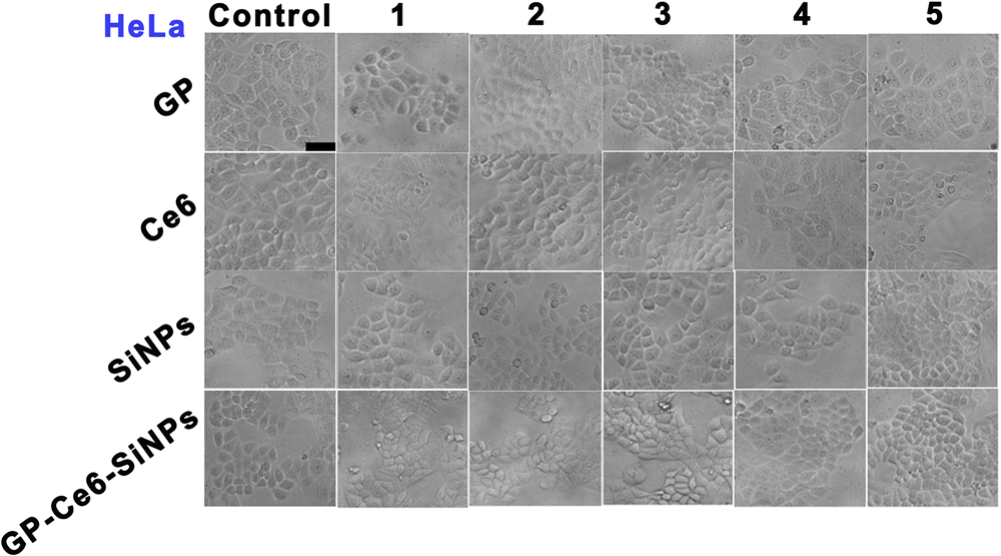


In Fig. 7c, the “Heart, GP-Ce6-SiNPs, (–)” image was a duplicate of the “Heart, PBS, (–)” image and both the “Lung, PBS, (-)” and “Lung, PBS, (+)” images were duplicates of the “Lung, GP-Ce6-SiNPs, (+)” image. The corrected Fig. 7c can be found below as Fig. 2

Fig. 2 Corrected Fig. 7c.
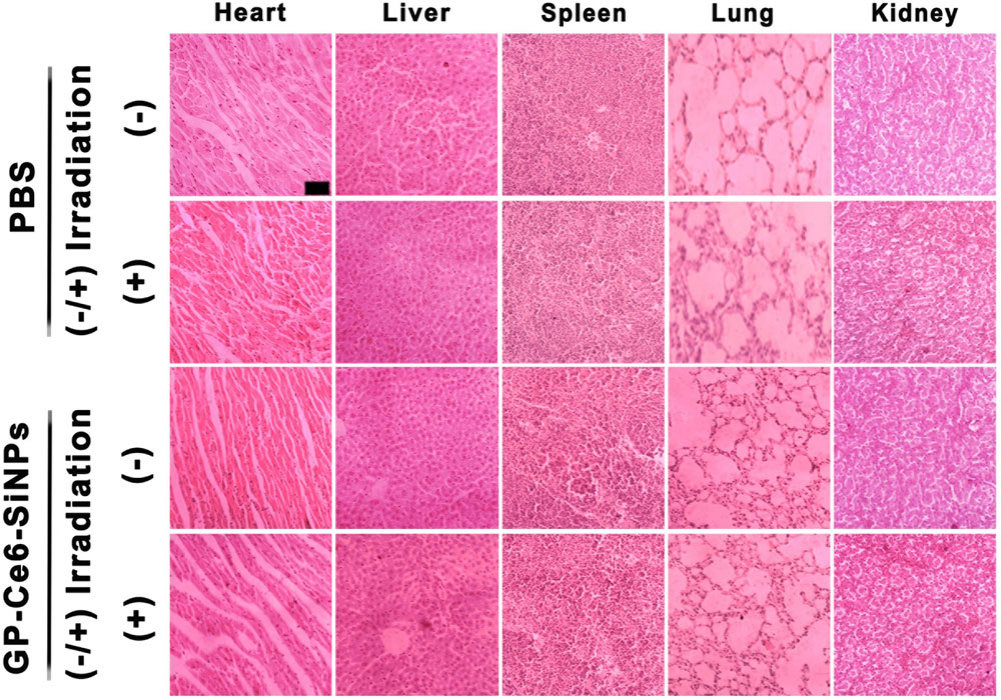


In Supplementary Fig. 6, the “SA, DIC” image was a duplicate of the Supplementary Fig. 9 “SA, DIC” image. The corrected Supplementary Fig. 6 can be found in the accompanying Supplementary information. This notice serves to amend these errors.

## Supplementary information

Updated Supplementary Fig. 6

