# Supplementary figures and images for: Author Correction: Multifunctional nanoagents for ultrasensitive imaging and photoactive killing of Gram-negative and Gram-positive bacteria
Source: Nat Commun. 2025 Jul 17;16:6582. doi: 10.1038/s41467-025-61902-y (PMC12271564; doi:10.1038/s41467-025-61902-y)

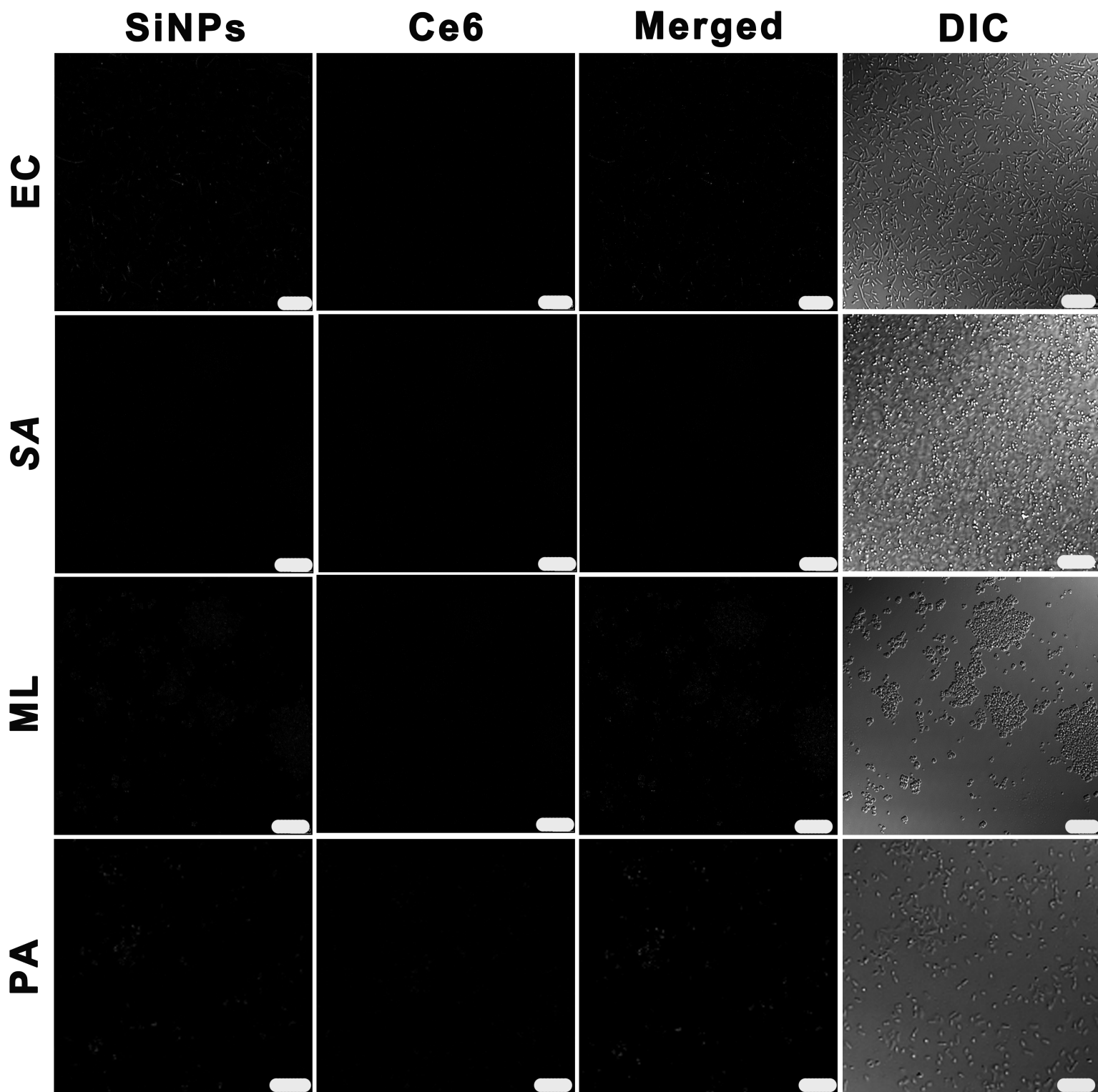

Supplement: Supplementary file 1 — Updated Supplementary Fig. 6 [file 41467_2025_61902_MOESM1_ESM.pdf]
